# Integrating Social Dimensions into Future Sustainable Energy Supply Networks

**DOI:** 10.3390/ijerph17176230

**Published:** 2020-08-27

**Authors:** Matevz Obrecht, Yigit Kazancoglu, Matjaz Denac

**Affiliations:** 1 Department for Sustainable Logistics and Mobility, Faculty of Logistics, University of Maribor, Mariborska Cesta 7, 3000 Celje, Slovenia; 2 Department for International Logistics Management, Yasar University, Izmir 35100, Turkey; yigit.kazancoglu@yasar.edu.tr; 3 Department for Technology and Entrepreneurial Environment Protection, Faculty of Economics and Business, University of Maribor, Razlagova 14, 2000 Maribor, Slovenia; matjaz.denac@um.si

**Keywords:** energy supply network, public perception, social integration, sustainable energy, supply chain management

## Abstract

Environmental protection and sustainable development have become an inevitable trend in many areas, including the energy industry. The development of energy supply networks is strongly correlated with the economics of energy sources as well as ecological and socio-political issues. However, the energy supply network is often distant from the social perspective. This paper therefore combines examination of perceptions and awareness of general public (web-based questionnaire) and top energy experts (a Delphi survey) on the energy supply network and identifies their potential integration in energy supply decision making processes. The results showed that public should be better informed as well as integrated into designing energy supply network as the prosumers gain power and the energy suppliers will no longer dominate the market. Public actors are ready to shape sustainable energy supply and also willing to pay 5.8% more for a sustainable energy supply. The majority are prepared to invest in renewable energy supply network close to their place of residence. Another result is that the public is calling for a shift in priority towards more sustainable and socially friendlier energy supply rather than focusing mainly on the economic and technical perspectives.

## 1. Introduction

Energy supply is essential prerequisite for human life, its well-being and sustainability for present and future generations. In this respect, United Nations Sustainable Development Goal no. 7 calls for ensuring “access to affordable, reliable, sustainable and modern energy for all” [1]. Enabling a secure energy supply, improving the efficiency of the energy supply chain and increasing the share of renewable (sustainable) resources in the national energy mix require the implementation of new business models, which ideally should involve various stakeholders—energy experts, but also the general public—among whom prosumers will be key actors in future energy supply chains [2]. Macroeconomic and social characteristics of each country structurally influence the sustainability and resilience of the energy supply-chain [3,4]. So far however, the literature has focused mainly on economic and environmental considerations and to a much lesser extent on social sustainability. As a key problem of efficient and fast realization of energy projects underestimated and diminished integration of social dimension is identified. Consequently, negative public perception on some renewable energy projects may reduce ability to mitigate climate change. Not only can social sustainability practices help to improve other aspects of sustainability, all three dimensions are needed to build a truly sustainable supply chain [5,6]. If social dimension is considered to a higher extent, opposition and undermining of energy projects (wind farms, river dams, new power lines etc.) from different stakeholders can be minimized. Public initiatives with a goal to stop energy projects are usually seen as an emergency tool for those stakeholders that were not addressed in project preparation. As best seen from the case of the Danish island of Samso, successful transformation to energy self-sufficiency based on sustainable energy sources required time-intensive negotiations with the local community and was achieved by successful integration of the local stakeholders as strategic and financial partners of the energy industry responsible for the new energy production facilities [7,8].

The social dimension should therefore gain priority in shaping future decisions on energy supply networks, energy policy and related climate change mittigation. Yet, due to the lack of integration of the social dimension in general, data based on the social dimension is not sufficiently explored. Main research question is therefore based on how to evaluate public perception and assessment on energy policy priorities, energy sources and energy projects as well as their acceptability and of course to see which data to use and how to integrate them into potential public-private partnerships. Adding social dimension data hotspots could enable data-based decision- making in a sustainable energy supply network.

### 1.1. Energy Supply Chain Priorities—Struggling away from Public?

Nowadays energy supply chains mainly focus on a top-down approach where decisions are influenced by strong energy organizations and energy lobbies. Public interests are therefore heavily underweighted and neglected. As a result serious conflicts may arise between energy producers, distributors and public interests, which may limit the transition to sustainable energy [9]. Both public opinion and consumer behavior cannot easily be taken into account when designing sustainable energy supply chains [10]. The consideration of the consumer perspective usually bridges socio-technical dynamics and equity aspects of sustainable energy transitions (e.g., dealing with energy poverty). In existing practices, however, these are mainly treated as separate phenomena [11]. Sometimes the full integration of public interests in the energy supply chains is contestable or even not advisable because the public is not so well informed about energy issues, challenges and constrains (financial, technical, etc.). Supply chain designers rely more on the assessment of energy experts than on public opinion as experts have detailed information on energy issues. Even though, in order to find out to what extend public opinion and experts assessment are (in)uniform, both should be examined and compared. New approaches and initiatives to involve different stakeholders in the energy production and distribution processes by implementing different cooperation mechanisms with different opinions can clarify the roles of individual participants in advance, which can also have positive impact on energy consumption [12,13]. Taking into account a consistency analysis of general public perception and energy experts’ assessment of key energy issues could help to provide better insight into actual public knowledge on energy related issues.

### 1.2. Increasing Importance of Social Dimension Data

The social perspective, including public perceptions of energy issues, is becoming increasingly important, so it is necessary to consider identifying and monitoring the social acceptability of energy policy and public opinion on various measures taken within energy policy strategies [14]. It is repeatedly proven that the social dimension is understudied [15] and that in the changing environment of increasing consumer power and the need to move to new business models and new supply chains, focused on pro/consumer. When talking about the transition to sustainable energy, it is necessary to raise public awareness of energy issues and to integrate public participation in energy policy is necessary but the first prerequisite is the collection of sufficient data. With the development of decentralized energy supply based on renewables, smart grids and inter-connectivity (e.g., Internet of Things) huge amounts of data have to be collected and processed to enable efficient system functioning. However, it is not yet clear which data will have to be collected, how they can be monitored, who will use them and in what way, as in the case of performance and economic data. They will be actively involved in energy production, distribution and use and will provide different feedbacks to energy companies [16,17] so their role will become more important for the redistribution of “power” in energy systems.

Lund describes that awareness of the choice is crucial to achieving 100% renewable energy solutions but, for this to happen stakeholders must first obtain data on different alternatives. He believes that the key driver for an efficient transition to sustainable energy production, distribution, storage and use is public awareness of different alternatives (choice awareness theory). The advantages and disadvantages of each alternative solutions should be presented and discussed with different stakeholders, as the energy producers and distributors who actually shape and implement energy policy are no longer just a few large energy companies but also a growing number of small prosumers and small and medium sized enterprises (SME). This required additional data background and data sharing but energy projects were consequently better accepted and therefore more efficient and socially more appropriate [18,19].

### 1.3. Aim and Scope of the Study

However with regard to the reviewed literature, a lack of information on data management to promote sustainability in energy supply chains was identified. To achieve sustainable supply chain management (SSCM), economic, environmental and social perspective should be considered together [20]. Data on the social dimension appear to be particularly undervalued, although they are about to become one of the foundations of future sustainable energy production, supply and distribution and we believe that this causes problems with efficient and fast realization of energy projects Consequently, negative public perception on some renewable energy projects may reduce ability to mitigate climate change.

Partners in the energy supply chain should find out what society thinks about energy issues, future priorities, their integration in the decision making process as well as their involvement in the energy sector. The apparent depletion of resources and the continuing pollution have led institutions, producers, suppliers and consumers to change the way they produce and consume energy [21,22,23] from an environmental perspective. To make it truly sustainable, it must also be socially sound.

This paper aims to assess the degree of integration of society in development of sustainable energy supply networks. The first step is the definition and analysis of general energy supply chain as well as its specific variations in relation to different energy sources and consequently the supply network. With regard to energy supply chains, current data integration, hotspots and potentials for increasing public participation can be defined. The study focused on the perspectives of stakeholders (general public and energy experts) on various current and future energy issues is structured as follows:

(a) Definition of energy supply chain variations and energy supply network;
(b) Integration of data into energy supply network planning;
(c) Awareness and participation of stakeholders with particular emphasis on potential public-private partnerships in local, national and global energy projects;
(d) Priorities for a sustainable future energy supply network;
(e) Sustainability assessment of the current and potential future energy supply network
(f) Sustainability assessment of energy sources;
(g) Synthesis of the results to propose the integration of various additional data hotspots to enable data-based decision making in the design of a sustainable energy supply network.

## 2. Materials and Methods

The analysis of energy supply chains was compiled on the basis of the literature review and critically analyzed to identify differences and specifics of the different energy supply chains that form an electricity supply network. Data based on the integration of social dimension in a sustainable future energy supply network were collected by means of a highly structured web questionnaire. The subjects studied were:

(a) General perception of the energy supply network and energy policy;
(b) Stakeholder participation in a sustainable energy supply network;
(c) Priorities for a sustainable future energy supply network;
(d) Sustainability assessment of the current and potential future energy supply network and
(e) Sustainability assessment of different renewable and conventional energy sources.

The population group was Slovenian public aged between 18 and 62 years (defined as active population). However, the focus was especially on younger generation with higher education since they are likely to lead the way and be key actors in the transition to a more sustainable future. The sample of this survey comprised 453 respondents, mainly aged up to 40 years. The largest age group was people aged 20–30 (63.6%) followed by 30–40 (16.7%). 42.7% of the respondents were men and 57.3% women. Within the general public we have separated lay and professional public. The professional public was identified as part of the respondents who work in the energy sector or have formal education/training in the energy field and therefore have more information on energy issues (Table 1). Results are presented separately only in case of c) Priorities for a sustainable future energy supply network since no significant differences among lay and professional public were identified in other cases.

In addition these data were compared with data from top energy experts who assess energy situation and the energy future in a multi-stage Delphi survey. These data were used to analyze the consistency of public opinion and the assessment of top energy experts. Firstly, the top energy experts eligible to participate in the study were identified (56 experts) and selected according to their professional experience, job title, description and field of activity (e.g., government, private enterprises, scientists etc.) and length of employment in energy sector (above 10 years), education, participation in energy projects and publications in the energy sector in professional and scientific journals. These experts were contacted personally, informed about the topic and the procedure of the Delphi survey and invited to participate in the survey (the first stage—answering the questionnaire lasted app. 30–45 min). The Delphi survey with a predominantly structured questionnaire was conducted over a period of 8 months. The data from the first Delphi round and its interpretation were then made available to the participating experts (20 experts), who could further shape their opinion in the second phase of the survey to improve the validity of the results. Due to the specifics of the Delphi study, the data on the forecast of sustainable energy development were published separately in Obrecht and Denac (2017, [24]). This study however contains original data on the sustainability of energy supply networks that have not yet been published.

The collected data were analyzed with descriptive statistics of qualitative data (shares, averages etc.). The Likert Scale (interval from 1 to 5) was used to assess the relevance of selected topics examined. The results of both studies were compared to assess consistency of public opinion and experts’ assessment on sustainable energy supply issues and integration of missing data into energy supply network design a planning.

The synthesis of findings is than gathered to identify current energy supply network focus and gaps (presented in Section 3.2) and to propose integration of sustainability perspective with greater focus on lacking social dimension data (public perception, social acceptability, public opinion) as proposed by the public and energy experts (Section 3.6).

## 3. Results

### 3.1. Defining Energy Supply Chain Variations and Energy Supply Network

**Figure 1:**
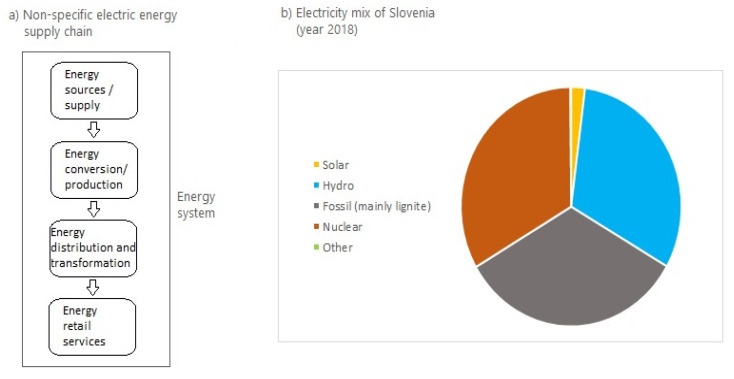
(**a**) Electric energy supply chain and (**b**) electricity mix in Slovenia (source: SURS, 2019 [31]).

Energy supply chains have undergone radical changes during their evolution [25]. The energy networks of the 21st Century in the EU focus on increasing cross-border transmission capacity, resilient energy supply and reducing their environmental impact and are therefore becoming increasingly complex. Sustainability improvements are mainly achieved with integration of cleaner technologies, renewables, decarbonization, increasing energy efficiency and ensuring sustainable energy use. Studies using supply chain models in energy systems primarily pose the challenge of how the energy supply and demand side can be systematically improved, taking into account environmental sustainability and efficient economic performances—while minimizing costs [3,26,27,28], but also to increase the social sustainability of energy supply. Even if the energy supply chain can be simplified as shown in Figure 1a, different energy sources are based on different specifics and form different energy supply chains with different steps in energy conversion process [26]. There is therefore considerable scope for improving the modeling process of efficient supply chain design and operation [29,30]. In terms of specific energy supply chains and connections with distributors and retailers, and in the near future with prosumers, the energy supply network is increasing its complexity.

**Figure 2:**
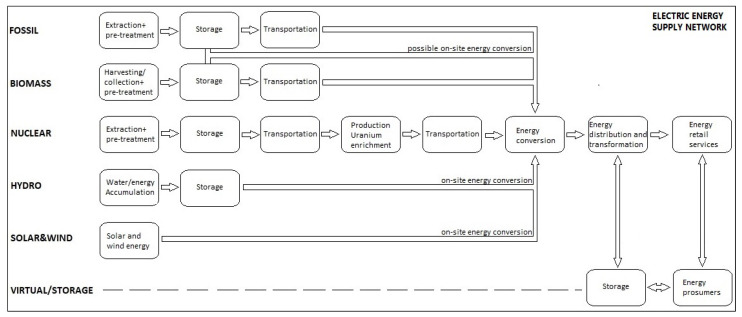
Different energy sources supply chains’ pre-production specifics (sources: Saavedra M. et al., 2018 [3]; Cucchiella & D’Adamo, 2013 [20]; Nunes et al., 2020 [29]; Mafakheri & Nasiri, 2014 [32] and own data).

As can be seen in Figure 2, different energy sources have different processes of converting primary energy into electricity. With regard to the process phases within the supply chain, solar&wind and hydroelectric power plans are the most promising, which allow energy conversion on site without “resource transport” and in case of solar&wind also without (pre)storage (in case of hydro power plant, on-site water accumulation can be identified as “primary energy storage site”). This can also be seen as a disadvantage if the goal of achieving stable and constant electricity production is pursued, which is not only possible with solar or wind power plants. On the other hand, nuclear energy has the most complicated process and the highest number of operations within the energy supply chain, but it is also the most stable and constant and therefore suitable for balancing supply and demand in the “not-smart” grid. It also represents a significant part of the Slovenian electricity mix (Figure 1b).

Including both horizontal (supplier-supplier) and vertical (customer-supplier) relationships, sustainability in the energy supply network can be achieved not only by focusing on cleaner technology solutions, but also by changing habits and transforming particularly vertical relationships into a cooperative partnership.

In terms of the development paradigm, the EU’s energy supply networks will mainly move towards more sustainable options. The inclusion of prosumers is therefore necessary, but not yet so common and is rarely applicable (partly only in retail energy trading). Due to the future demand for data-based energy supply chains to balance energy supply and demand and the associated integration of vertical relationships, the involvement of different stakeholder in different parts of the energy supply chain—resource extraction, power generation, electricity distribution (e.g., electricity grid operators), electricity retail and SMEs—will become increasingly important in shaping the future energy supply network.

### 3.2. Fokus Energy Supply Network Planning Data

Approximately 1900 local energy systems were developed by companies and communities globally. At present, these still dominant complex centralized networks based on fossil fuels will be forced into a transition process due to various natural, technological and social developments and constrains, such as increasing demand, economies of scale, geopolitical vulnerability of energy infrastructure and resource complementarians as well as depletion and climate change [33,34]. Accordingly, the energy system in many countries of the world is undergoing change [4] due to rapid technological and institutional changes at both central and local level towards a combination of top-down and bottom-up systems. As a result database based management systems are receiving considerable attention as the need for energy-related data increased even before 21st century [35]. No matter that decision making in the energy supply networks is based on numerous datasets that can be divided among technical and managerial datasets only (see Table 2). Core data collection and management is seen in the forecasting of electricity demand and supply in order to predict future electricity prices and to manage the balancing of excess electricity with electricity shortages, which will become even more important with a higher share of dispersed renewables [36] and will become one of the main challenges for smart grid development as well as enterprise competitiveness [37]. No matter that, environmental and social dimension data are identified as a hotspot of missing data, urgently needed to design sustainable energy supply network.

Transition focused towards a low-carbon, co-operative smart decentralized system in developed and developing countries will enable much higher engagement of citizens and local communities [33,43,44]. Companies included in energy supply network management therefore need to put more attention on social dimension (public opinion and perception as well as on social acceptance) and develop new business models that at least partly considers its integration.

Shaping clean energy supply networks is a highly important topic from consumer perspective. Over 60% of study respondents evaluated it as a highly important topic with the highest grade on Likert scale from 1 to 5 (AVG = 4.49; Modus 4.80—general public and 4.85 professional public) which is more than evaluation made by energy experts in Delphi study [24]. Results proved that in general the public is not sufficiently informed, let alone included in energy policy and energy-related projects that sometimes concern a high share of the general public or even are of national importance.

While emerging modes of energy supply networks will be consistent of environmental and social dimension data, this is yet defined as a hotspot to include in the sustainable energy supply network design. No matter that existing energy technology and energy project economics data should not be avoided in the future, however to make in more transparent they should become available for interested stakeholders—final energy users and prosumers.

### 3.3. Stakeholder Perception and Participation: Towards Sustainable Energy Supply Network

In general, the public would like to get much more information about investing in renewables (57%), especially in solar energy, which is becoming more interesting again because of changed energy policy, direct on-site conversion and shorter supply chain, established subsidizing schemes and net-metering in many countries within the EU. Despite the high relevance of the studied topic the lay public is not well aware of energy supply chains or even energy policy directives and related investments. 63% of them believe that they are “very badly informed or even not informed at all”. Only 22% of them—mainly professional public—declare that they are well informed about energy issues. In general, the public was mostly aware only of the controversial investment in a new block of a lignite-fired powered plant which was a popular media issue from the decision for its construction till the present when investment irregularities were detected.

A social dimension-lacking approach was identified when analyzing best energy practices in local as well as projects abroad (52%); namely in national energy projects in progress (51%), lacking assessment of impacts of energy technologies on environment and society (44%) as well as possible energy efficiency improvements (42%) and cost benefit analysis of energy production including external costs (35%). Approximately one third of respondents stated that they miss better data support on alternative scenarios for selected energy projects. Due to integrating prosumers into future energy supply and demand planning is predicted, new business models for such integration will have to be developed. Along with specific data-based decision models based especially on data on energy demand and production patterns and forecasts, potential energy supply etc., for efficient data-driven approach of designing more sustainable energy supply chains, additional data will have to be included. These data are going to be based on increasing importance of integrating broader environmental data as well as including currently lacking social dimension data based on stakeholder integration and participation.

Results revealed that 93.9% of respondents want to be better informed about studied issues especially when energy projects are being realized near their place of living. Approximately a third (34%) of respondents would like to participate at the design phase if projects are realized close to their homes. Only 15% of respondents were not interested in integration and participation in energy supply. This means that consumer/prosumer friendly energy supply chains as well as energy policy are becoming the new normal and, high share of respondents believe that energy sector do not include them sufficiently, however, it is not clear why not. This could be an issue of lacking data or avoiding managing public participation. However, to correct this, data gathering schemes must first be established and data monitoring should be upgraded to data driven just-in-time energy supply.

Integration could be done in different phases and with different levels of participation. It is interesting that energy production and distribution companies exposed many times that energy projects are extremely expensive and financing can be challenging. On the other hand, almost 20% of participants of this study stated that they are interested to invest their capital into energy projects. More than half of them (50.4%) were interested in investing in local projects including renewables, increasing energy efficiency and especially applications of new technologies (e.g., smart grid, V2G, IoT, AI) (44.1% for national and 21.0% for global projects). Even if small private investors are seen as a small-scale financers (e.g., 1.5% of them already invested in PV power plant, 7.8% into roof integrated solar heating and 14.5% in heat pumps) their current investments are already redesigning national energy system and related energy supply chains. If these investments would be further developed they could easily multiply the effect of energy supply chain partners towards cleaner future energy supply while bringing additional benefits to both parties. Energy producers and distributors would get financing and service users (public stakeholders) would get relatively safe long-term energy investments (supported also by the national energy strategy and renewable energy financing scheme), not to mention higher support of cooperating local communities.

Future data-driven energy supply chain based on smart grid application supported by prosumers must go in both directions—it should enable better utilization of produced energy for energy distributors and producers, higher production efficiency and reduced energy losses. All of that could be reflected even as a lower electricity bill to motivate prosumers’ willingness to progress further into partnerships with other energy producers and distributors. Data sharing platform could be one such solution that would connect all stakeholders included into energy supply chain and enable them to optimize their business decisions based on wide data monitoring and big-data IT support. Establishing such platforms for sharing and selling e.g., excessive electricity produced in PV power plants in time of high solar radiation is seen as a bright step toward it.

Due to high interest in private-public partnership especially in case of investments in renewables, energy efficiency and new technologies as well as the fact that 83% of participants are willing to pay in average 5.8% more for environmentally friendlier energy, thus, public should not be released from designing future energy supply chains.

### 3.4. Priorities for a Sustainable Future Energy Supply Network

As a part of a better integration of social dimension into future energy supply network, public preferences for energy priorities were also investigated. Some relevant differences in priorities ranking between the general public, professional public and energy experts were observed. Recently, we have witnessed progressive expansion, e.g., of the circular economy and bio-economy as well as other similar concepts, developed and implemented with a goal to reduce environmental impact and enable creation of more sustainable future. Even though these concepts have proved to have significant benefits and they are becoming the scope of policy makers, scientific consensus is more in favor of “de-growth”, “efficient use” and “sharing” concepts [45]. Sadly, these concepts mainly do not gain such unremarkable political support and are therefore many times not seen as priorities. Ranking of preferences on energy supply priorities within three stakeholders’ perspectives are presented in Figure 2 and it can be seen that “de-growth” and “efficiency” are the most relevant for top energy experts but general public is not that much focus in “de-growth” but rather more in “environmental soundness”.

**Figure 3:**
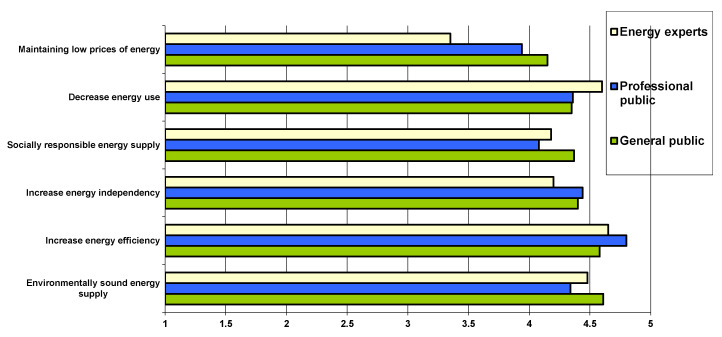
Energy supply priorities assessment by different stakeholders on Likert scale (1 means least important and 5 means most important.

As seen in Figure 3, it must be mentioned that despite the ranking, all priorities were evaluated rather high. “Increase energy efficiency” had similar importance for all three stakeholder groups. It is interesting that in case of two best evaluated priorities (AVG above 4.5) (“Planning environmentally sound energy supply” and “Increase energy efficiency”) consistency of general public assessment with top energy experts is actually higher than consistency of results of top energy experts and professional public. This was also true for third and fourth priority. Priority ranking with best evaluated priorities by general public (“Planning environmentally sound energy supply”, “Increase energy efficiency” and “Increase energy independency”) and least important priority (“Maintaining low energy prices”) can direct energy supply network towards energy future that public wants and is actually more sustainable. “Decrease energy use” was the most outstanding and much more important for experts and slightly more for professional than for general public. This might show that some priorities allow technological change to be implemented to improve energy sustainability and others such as “decreased energy use” require also changes in life style, which are much more challenging especially when it comes to potential prosumers’ comfort. Energy experts are also more aware of actual energy related issues, better informed and operate with more actual and sector specific data. “De-growth” strategy related to “decrease of energy use” in combination of “increased energy efficiency” should be seen as a viable solution for the clean future energy supply planning.

### 3.5. Sustainability Assessment of the Current and Potential Future Energy Supply Network

**Figure 4:**
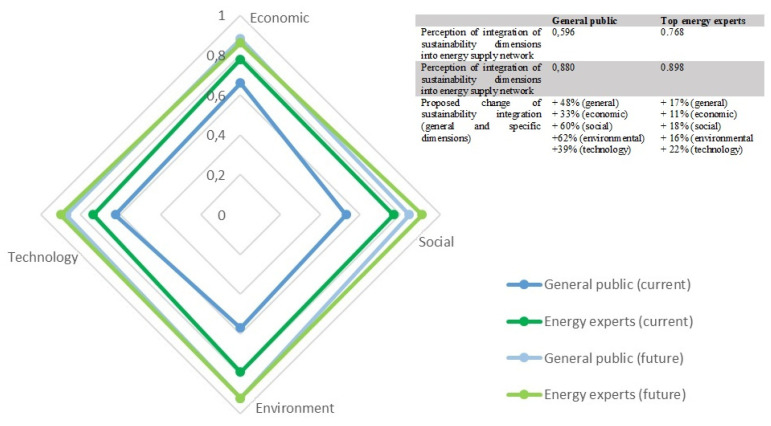
Sustainability assessment of current and future energy supply network.

Regarding the Slovenian electricity mix that is a combination of app. 1/3 renewables (mostly large hydro power plants), 1/3 from thermal power plants (mostly lignite) and 1/3 from a nuclear power plant it could be assessed as well-balanced system however defiance to lignite fired power plant is relentlessly repeating. Sustainability assessment made on the public perception of energy supply network confirmed that different aspects of sustainability should be incorporated more strongly. General public believes that more emphasize should be given to environmental and social dimensions. Even experts, who claim that different sustainability perspectives are to some extent integrated into energy policy, evaluate that stronger integration is still needed. The sustainability of current energy supply network is evaluated to be 0.596 by general public (lay and professional public were not presented separately due to high similarity of results) and 0.768 by energy experts respectively (0—not integrated at all, 1—completely integrated) (see Figure 3). A majority of the public believes that economic and technology perspective are most considered when designing energy networks and that environmental and social dimension data are lacking (the highest gap among current and proposed integration in future energy supply network is at environmental—62% and social dimension data—60%). Respondents actually evaluated the social perspective as the least integrated in energy supply network design, which is reasonable due to pretty low public integration in energy investments, energy projects and energy supply planning in general which should be improved in the future (see Figure 4).

These results are partially consistent with energy policy assessment made by top energy experts in the Delphi survey. Experts evaluated that environmental perspective is already quite well integrated however they agree that there is still room for improvement. They actually evaluate that all perspectives should be integrated with a greater extent however, gaps among current and proposed integration of different sustainability perspectives are significantly smaller than at public evaluations.

### 3.6. Sustainability Assessment of Renewable and Conventional Energy Sources

Perception of sustainability of different energy technologies was also observed. According to average value of responds the highest support was granted to solar and wind energy (Figure 3) which is in accordance with general opinion on energy production [13,14,18,44]; Both sources are believed to be the most environmentally friendly as well as socially appropriate. Nuclear energy was evaluated as the most economical which is surprising due to relatively low public support on nuclear energy especially after Fukushima Daiici disaster.

**Figure 5:**
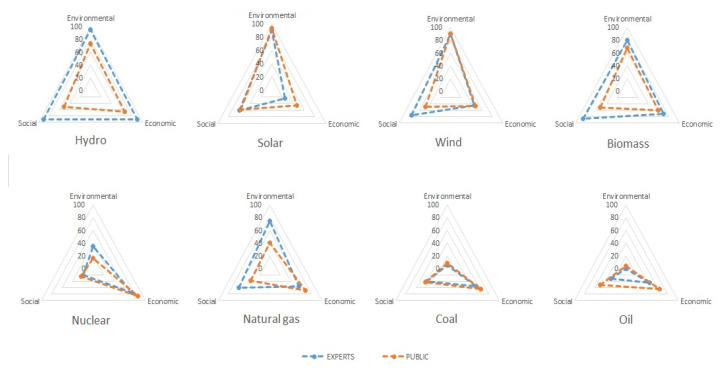
Public and experts assessments on sustainability perspectives of different electricity sources (environmental, economic and social dimension).

As presented on Figure 5, public perspective and experts evaluations differ significantly for different energy sources and for different selected dimensions of sustainable development. When evaluating appropriateness based on all three (economic, social and environmental) dimensions simultaneously, solar energy has the highest grades from general public (79.9%) and hydro from experts (91.9%). Solar and wind energy assessment from public and experts were most harmonized among renewables. On the other hand, hydro energy was surprisingly assessed by the public with lower environmental appropriateness than wind and solar energy. This was not consistent with the evaluation of top energy experts who evaluated micro and large hydro as the best technology for secure energy supply from sustainability perspective. The highest deviations among general public and energy experts among renewables were identified in social dimension which was assessed much lower in case of public assessments compared to experts assessment (hydro, biomass, wind and also partly solar).

In the case of conventional energy sources, the public evaluated social appropriateness of coal rather high due to discussion on the future scenarios of limiting and eventually closing practically new Slovenian thermal power plant with the highest GWP among all energy sources that would result in additionally limited jobs availability in that region. Among conventional sources natural gas was as expected identified as the most appropriate conventional energy source that is also socially acceptable according to the experts’ assessment. It can be seen as a transitional fuel enabling softer transition to sustainable energy future. Experts evaluated the environmental appropriateness of natural gas even higher, way above nuclear and similar to biomass/biogas. Here, the gap among social appropriateness assessed by the public and experts was not so clear. In general, coal was assessed as the least appropriate by public (nonetheless it is a local energy source in Slovenia as well as in Central Europe in general) and oil by the experts. It was however evaluated as currently relatively favorable from economic perspective since 65% of public and 55% of experts assessed it as economically feasible no matter that new future business models will have to include the polluter pay principle that may severely change coal’s economics.

### 3.7. Proposed Integration of Sustainability Data Hotspots for Future Sustainable Energy Supply Network: The Rise of Social Dimension Data

When summarizing the findings of integrating social dimensions into future sustainable energy supply network design and operation along with missing information (according to public perception and experts assessment), a focus on different data can be detected (Table 3). As presented in Table 2 emerging energy supply networks already include data on increasing production efficiency, purchase vs. sales price profit margin optimisation as well as forecasting and balancing electricity production and demand. If more sustainable energy supply network designs are to be pursued in the future, a shift towards stronger integration of sustainability perspectives divided into economic, environmental and social dimensions should be considered with social dimensions as a hotspot for efficient and fast implementation of future energy projects. Economic data are already well-integrated, however the future focus should be oriented towards comprehensive cost assessment, including external costs and considering life cycle perspective. Environmentally related data are also becoming the new reality due to increasing awareness, social need and legislation as well as the EU directives and New Green Deal framework. Social dimension significance is however not as highly valued and should become a key part of development of sustainable and data driven nationwide as well as European-wide energy network. The proposed data integration focused on social dimension data as well as on consideration of economic and environmental perspective, presented in Table 3, is extracted as a synthesis of findings of the study of public and experts assessment of energy supply network.

## 4. Discussion

International agreements and goals such as UN Sustainable Development Goals or Paris Climate Change Conference tend to reach an agreement on energy decarbonization, whereas the latter aims to combat poverty and social inequality while protecting the environment [44]. This challenge as well as consideration on energy issues is already a part of public perception of energy supply networks. There is also a desire for stronger public inclusion that leads towards the change of energy development paradigm. Consumer- or better prosumer-friendly energy development is becoming the new standard. Due to a high share of respondents in the general public segment as well as top energy experts believe that the energy sector does not include them sufficiently, stronger inclusion will have to be considered for future sustainable development in the energy sector resulting in integration of additional data for designing sustainable energy supply networks.

Energy supply is highly topical and interesting for a majority of the public (85%) and integrating their opinions and perceptions could bring additional benefits, especially in faster implementation of more controversial energy projects. In general, the public has a lack of knowledge and information about energy projects and energy policy development goals, which is normal and expected. However, they seem to have sufficient awareness of energy policy priorities and desire more knowledge on local energy projects being planned or realized. When discussing transparency in the energy industry, one third of respondents would like to get more information on alternatives for selected energy projects. This supports the conclusion that one third of people actually doubt that the best alternative was transparently selected and that the decision-making process can be questionable.

Even though more than half (50.4%) of them were interested in investing, especially in local energy projects (related with renewables and energy efficiency). Mumel et al. [46] came to similar conclusion for companies as well with special interest for solar photovoltaic. If such a high share of respondents is actually willing to invest their savings into energy projects, this is a valuable information for financing energy supply chains especially on a national but also on a regional level, since funds can be obtained by the local communities, private investors and companies. If case of public-private partnerships project related disapprovals and criticism are significantly reduced then, such projects can be realized with less complications. As an example, investments and construction of wind farms in Slovenia is completely stopped due to disapproval of local communities even though they can be defined as of high national importance. Best practices from abroad like Danish island Samso [47] or Austrian counties like Gussing or Weiz-Gleisdorf proved that integration of different stakeholders as well as public capital into energy projects brings multiplicatory benefits on the local and national level. Public participation on different levels is proposed also by different researchers [9,14,17,47] even though such supply chain is more complicated to manage and require better data support.

According to the survey results general public knowledge on energy projects was mostly focused only on the controversial investment in a new block of a lignite-fired powered plant. Opponents of different stakeholder integration are therefore critical to such social dimension integration especially due to lacking information and data available and absorbed by the public as well as because of sometimes misleading populism and mass media influence on the public perception like in case of Japanese nuclear power policy after Fukushima disaster [48]. Akash and Mohsen [49] actually discussed similar issue already back in 1999. They found out that if people believe that they only have certain energy sources available, they cannot be persuaded to use other, e.g., more environmentally friendly sources. Due to integrating prosumers into future energy supply and demand forecasting and planning is predicted [43], new business models for such integration will have to be developed. Along with specific data-based decisions, models based on demand and production patterns and forecasts will have to be expanded with additional data integrating triple bottom line perspectives. These data are going to be of increasing importance for integrating broader environmental datasets as well as including currently lacking social dimension data based on stakeholder perception, integration and participation and will in the future become important competitive advantage and value-added for companies in electricity production, distribution and electricity retail. More comprehensive databased decision making also enables reduced precipitation of manipulating with public perception as well as undermining of energy projects from general public. With appearance of inexpensive and easy to use data-based management systems, this can now be used not just by large enterprises but also by non-professional users like SMEs and prosumers with little assistance required from the programmer.

Evaluating energy supply network priorities with best ranked “Planning environmentally friendly energy policy” and “Increase energy efficiency” and least important priority “Maintaining low energy prices” can give energy supply chain managers and related decision makers a hint in which direction general public wants to move energy policy. Low importance of “Maintaining low energy prices” is also consistent with above commented results about willingness to pay 5.8% more for environmentally friendlier energy policy. Anyway, such a price increase must be studied additionally since it can also have a negative effect on energy poverty and standard of living, it gives clear sight towards how future energy supply network should look like—environmentally sound and socially fair. In combination with “decrease energy use” priority, highly evaluated by top energy experts that these priorities lead towards sustainable energy sector. There is however also the possibility that water use will be increased due to growth of renewables, that can however impact public acceptance of renewables [50]. Some studies argue that renewables are not as friendly to humans as to the environment but that they also have less impact per dollar-spent [51], so all of these can change priorities and acceptance of specific renewables in the future. The general public also favors renewable energy sources which can be identified as more expensive but of course environmentally more sound. There were differences among public and experts evaluations on energy sources which revealed that public perception might differ from the perception of energy experts. Even though, general directions of both stakeholder groups are similar—general public as well as top energy experts favor efficient, decreased and rational energy future as well as environmentally and socially friendly energy. Both groups also evaluate that sustainability should be stronger incorporated into energy policy planning and therefore they give strong support to renewables.

In addition, Slovenian as well as EU citizens could benefit from a more efficient and environmentally sound energy supply network where a substantial reduction in energy bills can be achieved [39]. Dispersed renewable energy sources also enable creation of more jobs especially in local SMEs [52] enabling countryside development.

Social marketing will however face major challenge when trying to ‘market’ reduced consumption to tackle climate change. Due to scarce resources, environmental and health impacts of energy industry as well as because of socio-political risks related with the Slovenian as well as general EU energy imports this will have to be done to reduce and adapt to the climate changes and it cannot be done without including public. Since social dimension is not sufficiently integrated into energy supply network design it should be reinforced in the future to avoid disruptions and enable development of sustainable energy systems. Therefore, supply chain managers developing sustainable energy network cannot proceed without databased decision making relying on: (a) external costs (environmental and social); (b) integrating social dimension data (perception of sustainable energy resources, production, distribution and use as well as integration of different stakeholders into energy supply design and planning); (c) cooperation with different stakeholders (e.g., local communities or private-public partnerships) as well as (d) comprehensive assessment of new technologies and concepts (e.g., IoT, AI, V2G, Smart grid, Smart communities, Industry 4.0 not just on their business but also on environment and society.

## 5. Conclusions

Data showed that inclusion of different public stakeholders into energy supply network design is lacking. Energy supply chains are based on data focused towards economic-efficiency, balancing supply and demand, and forecasting future electricity prices and do not pay enough attention to social issues like public perception, social acceptability and a cooperative society. The Slovenian public is poorly aware of the electricity supply network and its short- and long-term objectives. A majority would like to gain more information on selected and alternative energy projects as well as on possibilities to invest and cooperate in public-private partnerships.

Publically assessed energy policy priorities should be: (a) environmentally friendly energy policy, (b) increasing energy efficiency and (c) decreasing energy dependency especially from imported oil. Maintaining low energy prices is on the other hand not that important and majority is willing to pay extra fee for environmentally or socially friendlier energy supply. This is many times argued by energy distribution companies. Those that cannot guarantee carbon-free energy supply oppose this idea and those that can see it as a competitive advantage and a potential opportunity for adding value to their services. Even though differences among public and experts are clearly visible and it might also be true that general public has sometimes lacking knowledge on energy issues, both stakeholder groups have a clear vision of what the future energy supply should look like. These results are a valuable insight for energy supply chain managers as well as for designers of Slovenian energy supply network an energy policy directives. In electricity supply network with emerging smart grids, IoT and digitalization, it is highly recommended to include public perception, acceptance and cooperation into business decisions to avoid disruptions and to open the doors towards more sustainable and cooperative energy future. Another valuable conclusion is that, public investments into energy sector should be enabled especially in local energy projects related with renewable energy and increasing energy as a way to reduce the need for more expensive conventional financing and as a valuable leverage to decrease undermining of energy projects from public initiatives and excluded local authorities.

## Figures and Tables

**Table 1:** Stakeholder groups and data collection methods used in the survey.

Research Framework	General Public		Energy Experts
Stakeholder groups	Lay public	Professional public	Top energy experts
Data collection method	Web-based structured questionnaire		Delphi survey

**Table 2:** Integration of data in conventional energy supply network (unsustainable energy industry).

Perspective and Hotspots	Energy Sources Supply	Energy Extraction/Production	Energy Distribution	Energy Retail Services
Technical perspective	Resource availability/potential; Extraction/gathering technologies; Pre-treatment technologies; Extraction efficiency; Weather conditions and forecast.	Production efficiency & performance; Production potential; Power plant(s) maintenance intervals; Technology data; Radiation at nuclear power plant; Weather conditions and forecast.	P&D gap; Demand forecasts; Distribution losses; Network disruption replacements; Distribution technologies; Emerging technologies (e.g., IoT, AI, V2G, Smart grid, Virtual power plants, Smart communities, Industry 4.0)	Prosumers’ energy P&D; Industrial energy demand; Forecasting technology data; Emerging technologies (e.g., IoT, AI, V2G, Smart communities); Disruption forecasts—technical issues;
Managerial perspective (focus on economic datasets)	Resources’ prices; ROI on new resource extraction investments;	Forecasts & Energy demand; Production price including external costs; Primary energy price; Emission allowances price & quantity; Waste management costs; ROI on new electricity production investments; Scenario analysis;	P&D gap; Demand forecasts; Designing efficient and flexible energy supply; Costs of network operation; Energy price; ROI on new distribution and smart grid investments; Energy contracting; Electricity supply priorities; Scenario analysis;	Prosumers’ issues; Cost of balancing P&D; Forecasting P & D; Disruption forecasts—managerial issues; After sales financial considerations; Purchase & retail price (different time intervals); After sales costs; ROI on new electricity retail investments; Scenario analysis;
Sustainability gap—hotspots	Environmental data (assessing resource environmental sustainability); Social dimension data (energy sources social acceptance)	Partly environmental data (assessing and considering environmental issues of energy production); Social dimension data (energy production social impacts and acceptance)	Environmental data (assessing and considering environmental issues of energy distribution); Social dimension data (energy distribution social impacts and acceptance)	Environmental data (except from green marketing); Social dimension data (integration of prosumers and other stakeholders)

ROI = Return of investment. P&D = production and demand. Data extracted from: Saavedra M. et al., 2018 [3]; Dermont et al., 2017 [13]; Brandenburg et al., 2018 [15]; H. Lund, 2010 [18]; Obrecht & Denac, 2017 [24]; Zulkafli & Kopanos, 2018 [26]; Azevedo et al., 2019 [28]; Schroer & Goss, 1988 [35]; Pawar & Vittal K, 2019 [36]; Javied et al., 2019 [37]; Obrecht & Denac, 2013 [38]; Calise et al., 2018 [39]; Pawar et al., 2020 [40]; Guo et al., 2019 [41]; Said, 2003 [42] and own data.

**Table 3:** Integration of additional data for sustainable data-based energy supply network (data-based supply chain management) with focus on social dimension.

Data Integration	Energy Sources Supply	Energy Extraction/Production	Energy Distribution	Energy Retail Services
Economic data	Resources’ prices including external costs; De-growth strategies	Production price including external costs; ROI on new electricity production investments based on LCC; Public-private partnership (Soc-Econ)	ROI on new distribution and smart grid investments based on LCC; Public-private partnership (Soc-Econ)	ROI on new retail investments based on LCC; Public-private partnerships (Soc-Econ)
Environ. data	Resource environmental sustainability; Land use; Emissions (in air, water and soil); Social perception of sustainable energy sources (Soc-Env)	Radiation at nuclear power plant—impact on environment; Emissions (in air, water and soil); Primary energy use; Waste flows and circular economy; Social perception of sustainable energy production (Soc-Env)	Distribution losses (environmental perspective); Distribution related impacts; Potential environmental impacts of new technologies (e.g., IoT, AI, V2G, Smart grid, Smart communities, Industry 4.0)	Environmental assessment (“eco-labels”); Environmentally sound electricity, fuel, heat;
Social data	Noise (e.g., wind) Odor (e.g., biogas); Social responsibility at supply side; Resource sustainability perception	Noise; Impact on human health; Radiation (e.g., at nuclear power plant—impact on humans); Integration of stakeholders into energy supply network design and management; Cooperation with local communities	Distribution network design priorities (e.g., beneath/above the ground); Potential social impacts of new technologies (e.g., IoT, AI, V2G, Smart grid, Smart communities, Industry 4.0)	Educating and informing public—social responsibility of electricity, fuel, heat; Integrating social aspect into energy retail services;

Soc-Econ = Socio-economic issue, Soc-Env = Socio-environmental issue, P&D = production and demand, LCC = Life cycle costing, ROI = Return of investment.
